# Sarcopenia and Pleural Effusions: Exploring a Potential Link

**DOI:** 10.3390/muscles3030017

**Published:** 2024-06-22

**Authors:** Georgios I. Barkas, Nikolaos D. Karakousis, Zoe Daniil, Konstantinos I. Gourgoulianis, Ourania S. Kotsiou

**Affiliations:** 1 Laboratory of Human Pathophysiology, Nursing Department, University of Thessaly, 41500 Larissa, Greece; raniakotsiou@gmail.com; 2 Department of Respiratory Medicine, Faculty of Medicine, University of Thessaly, 41110 Larissa, Greece; 3 Department of Nursing, School of Health Sciences, University of Thessaly, 41500 Larissa, Greece

**Keywords:** sarcopenia, frailty, low muscle mass, pleural effusion, respiratory disease

## Abstract

Recent studies indicate a significant relationship between malnutrition, frailty, and pleural effusion (PE), highlighting the critical role of muscle mass in patient outcomes. This review investigates the association between sarcopenia—characterized by a decline in skeletal muscle mass and function—and PE, marked by fluid accumulation in the pleural space. The findings reveal that sarcopenia is prevalent in patients with PE and is linked to increased postoperative complications and mortality rates. In liver transplantation, esophagectomy, and lung cancer surgeries, sarcopenia exacerbates the risk of adverse outcomes. Notably, preoperative muscle mass assessment serves as a predictive tool for identifying patients at higher risk of complications. This review underscores the importance of early diagnosis and intervention for sarcopenia to improve clinical outcomes in PE patients. The therapeutic approach should include comprehensive nutritional evaluations and targeted muscle-strengthening interventions. By addressing sarcopenia, healthcare providers can significantly reduce PE-related complications, enhance patient recovery, and improve survival rates. This review provides a foundation for future research to develop effective strategies for the management and treatment of sarcopenia in the context of PEs, aiming to optimize patient care and quality of life.

## 1. Introduction

Sarcopenia, an age-related skeletal muscle disorder characterized by significant muscle loss and a consequent decline in body function, underscores the complexity of its diagnosis, which hinges on the presence of low muscle mass and function [1]. Various factors contribute to its development, including genetic predispositions [2,3], muscle underuse, and a decrease in motor units, underscoring the complexity of its causes [4]. The diagnosis of sarcopenia involves assessing muscle strength, quantity, and physical performance, yet lacks a universally accepted standard despite the availability of numerous diagnostic methods, including CT scans, muscle ultrasound, and the use of biomarkers [5,6]. This condition has garnered attention due to its association with increased risks of falls, frailty, hospitalizations, and higher healthcare costs, highlighting the variability of its prevalence based on age, gender, clinical conditions, and diagnostic criteria [7,8,9].

The consensus on diagnosing sarcopenia involves three criteria: low muscle strength, quantity or quality, and physical performance, with the SARC-F questionnaire serving as a crucial screening tool despite its limitations [6,10,11,12,13,14,15,16,17]. Additionally, other methods such as hand grip strength measurements, chair stand tests, MRI, CT, DXA, and Bioelectrical Impedance Analysis (BIA) are utilized for further detection and assessment, each with its advantages and drawbacks [6,16,18,19,20,21,22,23,24,25,26,27]. While there are no specific guidelines for sarcopenia treatment, interventions largely focus on training and exercise programs, particularly resistance exercise, and dietary adjustments to increase muscle mass and strength, highlighting the ongoing search for effective treatments and diagnostic tools [28,29,30,31,32,33].

Frailty is closely associated with sarcopenia and is characterized by diminished and/or incomplete recovery after numerous adverse events such as injury, infection, surgery, or emotional distress [18,34].

Pleural effusion (PE) represents the accumulation of fluid in the pleural space, a condition frequently encountered by respiratory physicians and associated with significant morbidity, mortality, and economic burden [35,36]. It is not a disease in itself but rather a manifestation of various underlying pathologies, such as increased pleural membrane permeability, pulmonary capillary pressure, decreased intrapleural and oncotic pressure, and obstructed lymphatic flow [37,38]. Patients typically present with symptoms like dyspnea, initially upon exertion, and pleuritic chest pain, varying with the fluid volume and underlying cause [36,37,39]. PE is a common complication in a range of diseases including congestive heart failure, liver cirrhosis, cancer, pneumonia, and pulmonary embolism, with its severity ranging from benign, as seen with viral pleuritis, to life-threatening when associated with malignancy or congestive heart failure [40,41,42].

Fluid in the pleural space accounts for 0.1 to 0.3 mL/kg and is typically filtered from systemic capillaries due to systemic vessels’ hydrostatic pressure [40,41]. The production and absorption of pleural fluid occurs on the parietal side of the pleura [41,42]. The fluid volume is controlled by the balance of pressure differences between the systemic and pulmonary circulation and the pleural space. This balance results in a small movement of fluid into the pleural space, which under normal conditions is then counteracted by lymphatic drainage [40,41,42,43]. The pathophysiology of PE involves an imbalance between pleural fluid production and reabsorption, influenced by hydrostatic and oncotic pressures between systemic and pulmonary circulations and the pleural space, leading to transudate or exudate classifications based on fluid protein levels [40,41,42,43,44,45,46]. Diagnosis and management of PE require a thorough clinical history, physical examination, and imaging with chest X-ray being fundamental, supplemented by chest ultrasound for better visualization of pleural septa [45,46,47,48]. The British Thoracic Society guidelines recommend diagnostic thoracentesis to ascertain the cause of effusion, with laboratory analysis of pleural fluid to distinguish between transudative and exudative effusions and guide further treatment [47,48,49]. This comprehensive approach is essential due to PE’s implication as a marker of advanced disease and its significant mortality rate among hospitalized patients [50].

Emerging studies have suggested a novel link between muscle weakness and the development of PEs, indicating that sarcopenia may play a role in the occurrence of this condition. In this narrative review, our objective is to delve into the potential association and interaction among sarcopenia, malnutrition, and frailty characteristics in relation to PEs. Given this preliminary evidence, our review seeks to explore and understand the dynamics between sarcopenia and PEs.

## 2. Materials and Methods

**Figure 1:**
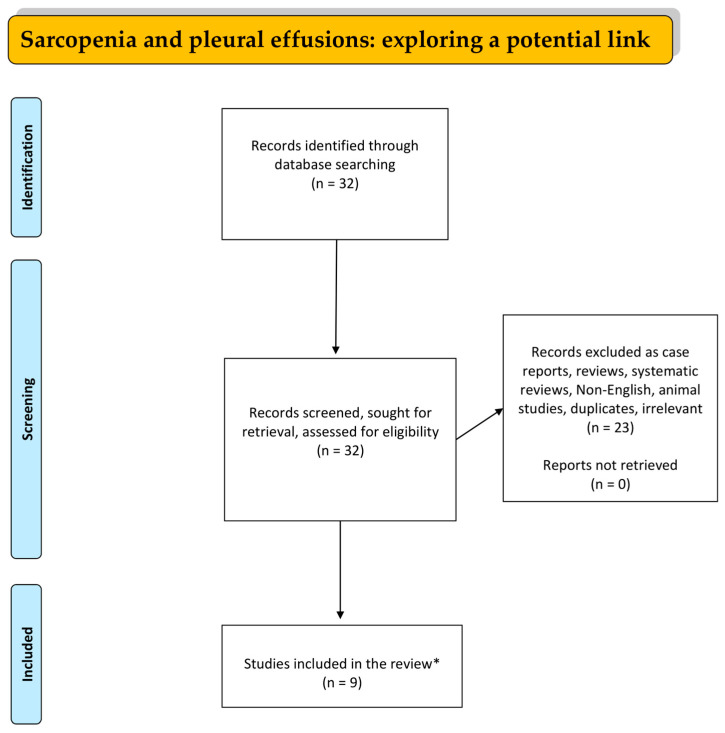
Diagram showing the literature review organization (* only original, written in English, non-animal research studies were incorporated in this review).

A literature search in the databases of PubMed, EMBASE, and Google Scholar was conducted from 1 October 1989 to 1 February 2024 utilizing the following combinations of specific keywords: “PLEURAL EFFUSION” AND “SARCOPENIA” OR “LOW MUSCLE MASS”. Only original studies written in English were incorporated into this study. Reviews, animal studies, children’s studies, irrelevant publications, case reports, letters, and comments to the editor were excluded. All the references of studies included were also investigated. The organization of the literature review is presented in the flowchart diagram (Figure 1).

## 3. Discussion

### 3.1. Sarcopenia, Pleural Effusion, and Liver Transplantation

Clouse et al. [51] explored the critical interplay between malnutrition and PEs in the context of liver transplantation. More specifically, they investigated how preoperative nutritional status, particularly the presence of malnutrition, which is often interlinked with sarcopenia, impacts the occurrence of PEs and subsequently influences early postoperative outcomes in liver transplant recipients. Only adult patients with radiographic evidence of PEs within 30 days before or after transplantation were considered eligible. During the four-year research period, 512 patients were recruited, with 107 (21%) developing peri-transplant PEs. Overall, 49 patients (10%) had a pre-transplant effusion, 91 (18%) had a post-transplant effusion, and 32 (6%) had both. The authors hypothesized that malnutrition, characterized by inadequate nutrient intake and altered metabolism, contributes to a weakened immune system and poor wound healing, factors that are critical for recovery post-transplantation. Additionally, it is supported that malnutrition can lead to alterations in fluid homeostasis, making patients more susceptible to developing PEs. The study suggested that malnutrition, a condition closely linked to sarcopenia, significantly contributes to the development of PEs, thereby exacerbating postoperative respiratory complications, prolonged hospital stays, and, in some cases, increased mortality rates among transplant recipients. Hence, the importance of comprehensive preoperative nutritional evaluations and interventions for patients awaiting liver transplants is emphasized. By identifying and addressing malnutrition and its associated risk factors, such as sarcopenia, healthcare providers can potentially reduce the incidence of postoperative complications like PEs, thereby improving overall patient outcomes and survival rates following liver transplantation.

Jain et al. [52] observed that sarcopenia, compounded by testosterone deficiency, could contribute to postoperative complications and functional decline in liver transplant recipients. Testosterone has anabolic effects that can help increase muscle mass and strength, potentially counteracting the effects of sarcopenia. Improved muscle function can enhance respiratory mechanics and cough efficiency, which are crucial for preventing atelectasis and facilitating fluid clearance from the pleural space. Therefore, while not explicitly stated in the study, the interventions explored by Jain et al. could indirectly reduce the incidence or severity of PEs by improving overall muscle health and postoperative recovery. Furthermore, the active in-bed exercises recommended as part of the therapy can aid in maintaining pulmonary ventilation and preventing fluid accumulation in the pleural cavity, further supporting the hypothesis that addressing sarcopenia and testosterone deficiency post-liver transplantation could have a positive impact on the management of pleural effusions. In conclusion, while the direct link between sarcopenia and PEs was not the focus of Jain et al.’s study, the exploration of testosterone replacement therapy combined with physical exercises to combat sarcopenia in liver transplant patients offers valuable perspectives on potential strategies to mitigate PEs. This approach highlights the importance of a comprehensive management plan that includes addressing muscle health to improve postoperative outcomes, including the prevention and management of PEs.

Wu et al. [53] conducted a study focusing on the relationship between sarcopenia and postoperative complications in living donor liver transplantation (LDLT) patients, particularly examining the impact of sarcopenia on the occurrence of major surgical complications, including PEs. By analyzing 271 LDLT recipients and utilizing the psoas muscle index (PMI) to quantify sarcopenia from cross-sectional images, the study sought to determine the predictive value of PMI for postoperative complications and explore how sarcopenia correlates with these outcomes, using regression analysis and ROC curve analysis. The study’s findings indicated a nuanced relationship between sarcopenia and postoperative complications, particularly noting gender differences in the impact of sarcopenia. For male patients, no significant association was demonstrated between PMI values and serious postoperative complications. However, in female patients, a significant correlation was observed; those with substantial postoperative issues had markedly lower mean PMI values (*p* = 0.028), with a defined cut-off value for sarcopenia at 2.63 cm^2^/m^2^. This suggests that sarcopenia, as measured by PMI, is a significant predictor of postoperative complications in female LDLT recipients. A critical finding of this study was the increased frequency of postoperative massive PEs requiring pigtail drainage in the sarcopenia group compared to the non-sarcopenia group (*p* = 0.003). This indicates a direct link between sarcopenia and the risk of developing significant pleural effusions post-LDLT, highlighting sarcopenia not just as a condition affecting muscle mass and function but also as a potential contributor to serious postoperative complications like PEs. Furthermore, the study explored the long-term survival rates based on sarcopenia status, revealing that female patients in the sarcopenia group had significantly lower overall survival rates at 1, 3, 5, and 10 years post-transplant compared to those in the non-sarcopenia group. This underscores the profound impact sarcopenia can have on long-term outcomes in LDLT patients, particularly among females.

In conclusion, Wu et al.’s study shed light on the critical role of sarcopenia, particularly in female LDLT recipients, as a significant risk factor for major postoperative complications, including PEs. The findings emphasized the need for preoperative sarcopenia screening, especially for female patients, to identify those at higher risk for postoperative complications and potentially tailor perioperative care strategies to mitigate these risks. Additionally, the study highlighted the importance of considering sarcopenia in the broader context of patient management and recovery, underscoring the potential benefits of interventions aimed at improving muscle health pre- and post-transplantation to enhance patient outcomes and survival rates.

### 3.2. Malignant Pleural Effusions and Sarcopenia

Rodríguez-Torres et al., in their study [54], explored the impact of sarcopenia on patients with malignant pleural effusions (PEs), focusing on the condition’s influence on symptom severity, overall health status, and hospitalization outcomes. They highlighted a clear relationship between muscle deterioration and the progression of PEs, suggesting that sarcopenia exacerbates the disease’s burden. The study employed DXA scans, CT imaging, grip strength tests, and gait speed tests to assess sarcopenia through muscle mass, strength, and physical function. Patients identified with sarcopenia were compared to non-sarcopenic counterparts in terms of health status and symptoms, using the EORTC QLQ-C30 questionnaire designed for cancer patients. The findings revealed that sarcopenia led to worse symptoms, poorer quality of life, longer hospital stays, higher rehospitalization rates, and increased mortality in patients with malignant PEs. These results underline the importance of muscle health in disease outcomes and patient prognosis. The study advocates for early sarcopenia screening and intervention, including nutritional support, physical therapy, and resistance training, to potentially improve the quality of life and clinical outcomes for patients. Additionally, it underscores sarcopenia as a modifiable risk factor in managing cancer-related conditions, emphasizing a holistic approach to patient care that addresses both the primary disease and its impact on patient well-being and survival.

Meggyesy et al. [55] conducted a retrospective study to explore the association between decreased muscle mass, indicative of sarcopenia, and survival rates in patients with malignant PE who underwent indwelling tunneled pleural catheter placement. They utilized computed tomography (CT) scans to estimate the cross-sectional areas of the pectoralis and paraspinous muscles as markers of muscle mass and aimed to correlate these measurements with overall survival. The cohort included 309 patients with a median age of 67, with females comprising 58% of the population. The study differentiated the impacts of Pectoral Muscle Area (PMA) and Paraspinous Muscle Area (PARA) on survival, conducting both univariate and multivariable analyses stratified by gender. Univariate analysis for PMA did not show significant differences in hazard ratios (HR) between high and low muscle areas, indicating no overall correlation between PMA and survival. However, gender-stratified multivariable analysis revealed a protective effect of higher muscle area on survival in males (HR = 0.63) but not in females.

For PARA, univariate analysis similarly showed no significant association with survival. Multivariable analysis suggested a potential protective effect of higher muscle area in males, though it was not statistically significant. The median survival from initial pleural drainage to death was noted to be 129 days. Overall, regression and Kaplan–Meier survival analyses did not establish a link between muscle mass and survival across the general population studied. A subgroup analysis of lung cancer patients highlighted that lower muscle mass correlated with shorter survival times, suggesting a pronounced impact of sarcopenia on survival within certain populations, such as those with lung cancer.

Meggyesy et al. concluded that while sarcopenia did not universally predict shorter overall survival among all patients with MPE, there was evidence of its adverse impact on survival in specific subgroups, notably males and lung cancer patients. This indicated a need for further investigation into the role of sarcopenia in malignant PE. The findings suggested that interventions aimed at preserving or increasing muscle mass could potentially improve outcomes for certain patient groups. The study underscored the nuanced relationship between sarcopenia and survival in malignant PE, pointing towards a gender-specific and disease-specific impact that requires deeper exploration.

### 3.3. Sarcopenia, Pes, and Cancer

Aro et al.’s study [56] provides a comprehensive analysis of the impact of sarcopenia and myosteatosis on colorectal cancer patients undergoing surgery, with a particular focus on postoperative respiratory outcomes and long-term survival. The retrospective study encompassed 348 patients, assessing skeletal muscle mass at the L3 level using venous phase computed tomography to classify participants as either sarcopenic or non-sarcopenic, and myosteatotic or non-myosteatotic. This classification enabled a detailed investigation into the correlation between these conditions and postoperative morbidity and mortality. The prevalence of sarcopenia was notably high, affecting nearly 60% of the study cohort, while myosteatosis was observed in approximately 31.2% of patients. The analysis revealed that sarcopenia significantly increased the risk of postoperative pneumonia and cardiorespiratory complications (such as PEs). Specifically, sarcopenic patients demonstrated a higher incidence of pneumonia (8.5% vs. 0.0%, *p* = 0.041) and cardiorespiratory issues (6.3% vs. 0.0%, *p* = 0.023) compared to their non-sarcopenic counterparts, with these complications more pronounced among patients with colon and rectal cancer, respectively. This suggested that sarcopenia not only affects surgical recovery but also poses a significant risk for specific postoperative complications. Additionally, the study highlighted the impact of sarcopenia and myosteatosis on patient discharge outcomes. Myosteatotic patients were notably less likely to be discharged home, indicating a greater need for institutional care post-surgery. This was also observed to a lesser extent in sarcopenic patients, suggesting that muscle degradation and fat infiltration within muscles adversely affect the ability to return to independent living postoperatively. In other words, Aro et al.’s findings illuminated the profound effects of sarcopenia and myosteatosis on both short- and long-term results for colorectal cancer patients undergoing surgery. The study conclusively demonstrates that these conditions not only elevate the risk of immediate postoperative complications, such as pneumonia and cardiorespiratory problems, but also predict the need for extended care and significantly impact overall survival. These insights emphasize the critical need for early identification and intervention strategies to address sarcopenia and myosteatosis in colorectal cancer patients, aiming to improve postoperative recovery and enhance long-term health outcomes.

Lee et al. [57] investigated the impact of skeletal muscle depletion on early postoperative complications in patients with early-stage non-small cell lung cancer (NSCLC) who underwent curative pulmonary resection. This retrospective study, spanning from 2016 to 2018, included 236 patients with pathologic stage I/II NSCLC. The researchers employed the psoas volume index (PVI), measured from preoperative positron emission tomography-computed tomography scans, as an indicator of skeletal muscle mass to examine its relationship with postoperative complications within 90 days of surgery. The study revealed significant differences between patients with low PVI and those with normal-to-high PVI in terms of age, body mass index (BMI), and psoas area index, indicating that patients in the low-PVI group were older, had lower BMI, and possessed reduced muscle mass. Furthermore, pulmonary function tests showed that the low-PVI group had significantly lower forced vital capacity (FVC), forced expiratory volume in 1 s (FEV1), and diffusing capacity for carbon monoxide (DLCO), suggesting compromised pulmonary function in patients with skeletal muscle depletion. A notable finding was the higher incidence of early postoperative complications in the low-PVI group (57.6%) compared to the normal-to-high-PVI group (32.8%), pointing to a significant association between reduced skeletal muscle mass and postoperative complications. While specific respiratory complications such as prolonged air leak and recurrent pleural effusion (PE) were more common in the low-PVI group, the differences were not statistically significant for these individual complications, including chylothorax, pulmonary thromboembolism, pulmonary infarction/torsion, pericardial effusion, and postoperative pneumothorax. Through univariate and multivariate analyses, the study identified significant risk factors for overall complications in the early postoperative period, including male sex, lower hemoglobin levels, smoking history, longer anesthesia time, and being in the low-PVI group. Additionally, risk factors for early postoperative respiratory complications, analyzed through both univariate and multivariate analyses, highlighted the PVI group, DLCO percentage, chronic obstructive pulmonary disease (COPD), smoking history, anesthesia time, and operation time as significant predictors.

In conclusion, Lee et al. found that skeletal muscle depletion, as measured by the PVI, significantly increased the risk of early postoperative complications, particularly respiratory complications, in patients with early-stage NSCLC undergoing curative pulmonary resection. This study emphasizes the importance of assessing skeletal muscle mass as part of preoperative evaluations to identify patients at higher risk for postoperative complications, including pleural effusions and pleural empyema, thereby guiding interventions to mitigate these risks.

### 3.4. Sarcopenia, Frailty, Pleural Effusions, and Mitral Valve Surgery

Ostovar et al. [58] conducted a study that explored the link between age, frailty, and postoperative complications, including PEs, in patients undergoing mitral valve surgery. This retrospective analysis included 1627 patients, segmented into three age groups: 40–59 years (n = 319), 60–74 years (n = 795), and over 75 years (n = 513), with individuals under 40 diagnosed with endocarditis being excluded. The objective was to uncover the reasons behind older age being considered a significant risk factor for adverse surgical outcomes. The researchers discovered that older patients were significantly more likely to suffer from pre- and post-operative renal insufficiency (*p* < 0.001), with pre-existing renal failure greatly increasing the likelihood of postoperative renal failure that required dialysis. Importantly, postoperative renal insufficiency was strongly correlated with the development of pleural or pericardial effusions (*p* < 0.001, *p* = 0.016), indicating a direct connection between renal health and these complications. Further findings indicated that patients over the age of 75 had significantly lower body mass index (BMI) than those aged 60–74 (27.3 vs. 28.2 kg/m^2^, *p* = 0.007). The study also noted an age-related increase in critical illnesses, such as myopathy and neuropathy (CIP/CIM), with a significant rise observed (*p* = 0.04). This increase pointed towards the presence of sarcopenia, defined by the loss of skeletal muscle mass and strength, a key marker of frailty, especially in older populations. Additionally, the study recorded a significant increase in both the length of hospitalization and mortality rates with advancing age (*p* = 0.013, *p* < 0.001). These results underscored the heightened vulnerability of older patients to severe postoperative complications and the potentially life-threatening nature of such outcomes. Ostovar et al.’s research concluded that older patients undergoing mitral valve surgery, especially those with advanced renal failure, faced a significantly higher risk of death, postoperative renal failure, dialysis, and complications such as pleural and pericardial effusions. The higher incidence of CIP/CIM and the reduction in BMI in the oldest patient group indicated sarcopenia as another critical aspect of frailty, in addition to renal failure.

In summary, their study highlighted the relationship between age, frailty, and postoperative outcomes among patients undergoing mitral valve surgery. It underscored the importance of thorough preoperative assessments to identify markers of frailty, such as sarcopenia and renal insufficiency, to better tailor postoperative care plans aimed at reducing the risk of pleural effusions and other severe complications in older patients.

### 3.5. Pleural Effusions, Sarcopenia, and Esophagectomy

In their study, Kemper et al. [59] delved into the association between computed tomography (CT)-derived muscle parameters and the clinical outcomes following esophagectomy, with a particular focus on the role of sarcopenia in the development of postoperative complications such as pleural effusions and pleural empyema. Analyzing a cohort of 98 patients who underwent esophagectomy, they assessed the skeletal muscle index (SMI) to gauge muscle mass and measured muscle radiation attenuation (MRA) to evaluate muscle quality. Their findings elucidated a nuanced relationship between muscle mass, as indicated by SMI, and the hazard of postoperative complications. Specifically, the study observed that an increase in SMI significantly reduced the likelihood of developing pleural effusions that required additional drainage procedures. The odds of encountering pleural effusions decreased dramatically, by −140.6% per 5-unit increment of SMI, highlighting a protective effect of higher muscle mass against this particular postoperative complication.

Moreover, the research revealed a similar protective trend regarding pleural empyema, a severe form of pleural effusion characterized by the presence of pus in the pleural space, suggesting that patients with higher SMI were less likely to develop this complication. The odds of pleural empyema decreased by −120.6% per 5-unit increment of SMI, further underscoring the beneficial impact of maintaining greater muscle mass in the context of esophagectomy. On the other hand, the study found that the odds of experiencing a pulmonary embolism increased with higher SMI, introducing a complex interplay between muscle mass and various pulmonary complications post-surgery.

Kemper et al. also explored the relationship between muscle parameters and the duration of hospital stays using a competing risk approach but found no significant association between muscle mass or quality and the length of intensive care or overall hospitalization. Furthermore, their survival analysis, employing the log-rank test and Cox proportional hazards regression modeling, initially indicated that lower MRA and SMI were related to decreased survival times. Nevertheless, this association did not hold after adjusting for confounders such as the Charlson Comorbidity Index, suggesting that factors beyond muscle mass and quality significantly influence long-term survival after esophagectomy. Consequently, Kemper et al. concluded that poor muscle status, as determined through CT imaging, should not deter patients from undergoing oncologic resection for esophageal cancer. They posited that the Charlson Comorbidity Index offers a more effective tool for preoperative risk stratification than muscle parameters alone, emphasizing the complexity of assessing surgical risks and patient outcomes. This study highlighted the protective role of higher muscle mass against developing pleural effusions and pleural empyema following esophagectomy, while also acknowledging the multifaceted nature of postoperative recovery and the importance of comprehensive patient evaluation. The main studies reporting on the connection between sarcopenia and PE are found in Table 1.

Across these studies, a compelling narrative emerges on the impact of sarcopenia and related conditions on surgical outcomes, particularly in the context of liver transplantation, esophagectomy, mitral valve surgery, and lung cancer resection. These studies collectively illustrate that sarcopenia, indicated by reduced skeletal muscle mass and quality, significantly correlates with increased postoperative complications such as PEs, and pleural empyema, as well as decreased survival rates.

Specific findings include the protective role of higher muscle mass against pleural complications post-esophagectomy, the nuanced relationship between muscle mass and PEs in lung cancer patients and lung cancer surgery patients, and the identification of sarcopenia as a critical risk factor for postoperative respiratory complications and overall mortality in liver transplant and mitral valve surgery patients. Notably, the research underscores the importance of preoperative muscle mass assessment and suggests potential benefits of interventions, such as testosterone replacement therapy, to improve outcomes. The diversity of surgical contexts covered by these studies highlights sarcopenia’s broad relevance as a modifiable risk factor deserving further investigation and clinical attention to enhance patient care and surgical success.

Throughout the studies conducted, several possible mechanisms emerge. Sarcopenia disrupts fluid homeostasis and compromises immune function increasing susceptibility to infections. Moreover, it weakens respiratory muscles impairing breathing and heightens the risk of respiratory complications post-surgery. Skeletal muscle depletion is associated with early postoperative respiratory complications. Sarcopenia reduces overall physical resilience and ability to manage fluid balance while older, frail, and sarcopenic patients are more prone to developing PEs post-surgery due to diminished muscle strength. Low muscle mass serves as a poor prognostic factor, indicating a higher likelihood of severe PEs. A higher skeletal muscle index (SMI) acts as a protective factor against developing PEs and pleural empyema post-esophagectomy by supporting better respiratory mechanics and fluid clearance.

However, studies examining the connection between sarcopenia and PEs face several limitations, including retrospective designs that challenge causality determination and small sample sizes that limit generalizability. The heterogeneity of study populations and lack of standardized methods for measuring sarcopenia and diagnosing pleural effusions introduce variability in the findings. Many investigations are single-center studies with limited geographic applicability, and there is often a lack of long-term data to assess enduring impacts. Additionally, the specific focus on particular surgeries may not represent the broader patient spectrum, and critical factors like nutrition and physical activity are frequently underexplored. Addressing these challenges is essential for advancing understanding and improving patient outcomes. Hence, further investigation and experimentation are required to understand the potential connection between sarcopenia and PE. It would be beneficial to conduct prospective studies to examine the impact of specific nutritional interventions on the occurrence of PEs in both pre- and post-liver transplant patients. Additionally, gender-specific research into how sarcopenia contributes to PEs in LDLT patients could provide valuable insights. Furthermore, studying the use of PVI as a predictor of respiratory complications in NSCLC patients, as well as assessing the impact of muscle mass on survival outcomes in patients with malignant PEs across different cancer types, can improve our understanding of sarcopenia’s role in PE development. Emphasizing pre-operative optimization is crucial, as evidence shows that sarcopenia increases the risk of postoperative complications. Therefore, pre-operative programs that focus on increasing muscle mass and improving muscle quality may effectively reduce the occurrence of PEs and enhance patient outcomes.

## 4. Conclusions

Recent studies highlight the significant linkage between sarcopenia, characterized by decreased muscle mass and strength, and the incidence of PEs in surgical patients. Sarcopenia has been shown to increase the risk of postoperative complications, including PEs and empyema, particularly in patients undergoing surgeries such as liver transplantation, esophagectomy, and lung cancer resection. The research suggests that sarcopenia’s impact on respiratory function and postoperative recovery may contribute to the accumulation of PF. Findings across various surgical contexts indicate that preoperative assessment of muscle mass could be crucial for identifying patients at higher risk for PEs. These insights underscore the potential benefits of interventions aimed at mitigating sarcopenia to improve surgical outcomes and decrease the likelihood of pleural complications.

## Figures and Tables

**Table 1:** The main studies reporting the connection of sarcopenia and pleural effusion.

Author/Ref.	Study Design	Study Population	Main Findings
Clouse et al. [51]	Retrospective study	A total of 512 LTs were performed.	A total of 21% of LT patients developed PE while PE was related to poorer outcomes across all clinical parameters. PE patients had a longer hospital stay (17 vs. 9 days, *p* < 0.001) and were more likely to be sent to a care facility (48% vs. 21%, *p* < 0.001). A total of 69% of effusion patients required a 90-day readmission, compared to 44% (*p* < 0.001). Patients with any effusion had a one-year survival rate of 86% (vs. 94%, *p* < 0.01).
Jain et al. [52]	Retrospective study	A total of 16 liver transplant recipients receiving posttransplant testosterone replacement therapy with functional sarcopenia were included.	Pleural effusions, as part of the overall body composition assessment, can influence the evaluation of sarcopenia in patients undergoing liver transplantation.
Wu et al. [53]	Retrospective study	A total of 271 LDLT recipients were included.	Postoperative massive PE requiring pigtail drainage performed more frequently in the sarcopenia group than in the non-sarcopenia group (*p* = 0.003). The 1-, 3-, 5- and 10-year overall survival rates in females were importantly poorer in the sarcopenia group (n = 14) compared with the non-sarcopenia group (n = 108), at 92.9% versus 97.2%, 85.7% versus 95.4%, 85.7% versus 92.5%, and 70.1 versus 82.0%, respectively (*p* = 0.041), and rates were 94.6%, 89.9%, 85.9%, and 78.5% in male patients. Sarcopenia is related to a significantly increased hazard of major postoperative complications in females.
Rodriquez–Torres et al. [54]	Observational prospective cohort	A total of 74 pts with MPE underwent measurements of symptoms, health-related quality of life, and functional status upon admission, discharge, and 3 months after hospital discharge.	Health-related quality of life and functional status were worse in subjects with MPE and sarcopenia, subjects with MPE and sarcopenia were symptomatic during hospitalization and at discharge. The distribution of pleural fluid location was comparable between both groups (93.75% vs. 80.95%). The duration of hospitalization was longer in the group with sarcopenia (14.69 vs. 10.84 days). Therefore, sarcopenia is a clinical feature with substantial negative effects in patients with MPE.
Meggyesy et al. [55]	Cross-sectional study	A total of 309 patients with MPE were available for analysis.	The presence of decreased muscle mass within a lung cancer population that has malignant pleural effusions was related to decreased survival. Multivariable analysis stratified by gender (female: HR = 0.81, 95% C.I. (0.57–1.16), *p* = 0.249; male: HR = 0.67, 95% C.I. (0.42–1.08), *p* = 0.101) suggests that higher muscle area, particularly in males, may have a protective effect on overall survival. Nevertheless, the presence of decreased muscle mass within a heterogenous population of malignant pleural disease was not related to decreased overall survival time.
Aro et al. [56]	Retrospective study	A total of 348 colorectal cancer patients were included.	A total of 208 patients had sarcopenia while 108 had myosteatosis. Sarcopenia was related to increased hazard of postoperative pneumonia (6.7% vs. 1.4%, *p* = 0.021). Sarcopenic colon cancer subjects had an increased rate of cardiorespiratory complications compared to non-sarcopenic (6.3% vs. 0.0%, *p* = 0.023), and sarcopenic rectum cancer subjects developed pneumonia more often than non-sarcopenic patients (8.5% vs. 0.0%, *p* = 0.041). Sarcopenia increases the pneumonia and cardiorespiratory complication rates including PE.
Lee et al. [57]	Retrospective study	A total of 236 patients with pathologic stage I/II NSCLC who underwent curative pulmonary resection were eligible and included.	Sarcopenia, as represented by a low PVI in this study, was demonstrated as a negative prognostic factor for overall early postoperative complications. Respiratory complications included prolonged air leak (16.9% in the low-PVI group vs. 9.6% in the normal-to-high-PVI group (*p* = 0.125)) and recurrent PE (11.9% in the low-PVI group vs. 6.8% in the normal-to-high-PVI group (*p* = 0.267)). Results confirmed an important correlation between sarcopenia and impaired pulmonary function recurrent pleural effusion was also more frequently observed in the low-PVI group.
Ostovar et al. [58]	Cohort study	The study enrolled 1627 patients who underwent mitral valve surgery. Patients younger than 40 years who had been diagnosed with endocarditis were excluded.	It appears that elderly subjects with advanced renal failure have a significantly increased risk of mortality, postoperative renal failure, need for dialysis, and possibly the development of pleural and pericardial effusions in mitral valve surgery. Furthermore, the proportion of pleural effusions and pericardial effusions was importantly higher with aging (*p* < 0.001 and *p* = 0.016, respectively).
Kemper et al. [59]	Observational study	A total of 98 patients undergoing esophagectomy.	No relevant association to lengths of stay in intensive care or hospital was recorded. If the SMI increased, the odds for pleural effusion and pleural empyema decreased, but the odds of a pulmonary embolism increased. Univariate, unadjusted long-term survival analysis demonstrated that decreased MRA and lower SMI were associated with shorter survival (*p* = 0.03). However, if the analysis was adjusted for confounders, e.g., Charlson Comorbidity Index, no relevant association regarding long-term survival was detected.

Abbreviations: LT: liver transplant; PE: pleural effusion; MPE: malignant pleural effusion; LDLT: living donor liver transplant; CT: computed tomography; ICU: intensive care unit; SMI: skeletal muscle index; MRA: muscle radiation attenuation; NSCLC: non-small cell lung cancer; PVI: psoas volume index.

## Data Availability

The data presented in this study are available upon request from the corresponding authors.
